# Prognostic evaluation of boys with posterior urethral valves using a recursive feature elimination machine learning framework

**DOI:** 10.3389/fped.2026.1906615

**Published:** 2026-09-08

**Authors:** Emilie G. Jaroy, Live Lundar, Gabriel T. Risa, Inger Nina Farstad, Gunnar Aksnes, Ragnhild Emblem, Rune Ougland

**Affiliations:** 1 Department of Pediatric Surgery, Oslo University Hospital, Oslo, Norway; 2 Faculty of Medicine, Institute of Clinical Medicine, University of Oslo, Oslo, Norway; 3 Alexion, AstraZeneca Rare Diseases, Av Diagonal, Barcelona, Spain; 4 Department of Pathology, Oslo University Hospital, Oslo, Norway; 5 Department of Surgery, Baerum Hospital, Vestre Viken Hospital Trust, Gjettum, Norway

**Keywords:** posterior urethral valves (PUV), bladder dysfunction, pediatric surgery, prognosis prediction, renal function, machine learning framework, recursive feature elimination

## Abstract

**Objective:**

To improve prognostic prediction and identify key predictors of renal and bladder outcomes in boys with posterior urethral valves (PUV) using machine learning (ML).

**Study design:**

Single center study of boys with PUV. A bespoke ML framework incorporating recursive feature elimination, supervised learning models, and 5-fold cross validation was developed for prognostic prediction and identification of key features.

**Results:**

Patients diagnosed after one year of age (*n* = 22, late diagnosis group) had normal renal function at last follow up. Renal outcome analysis was therefore focused on the 83 patients diagnosed before one year of age (early diagnosis group), of whom 22% (18/83) developed chronic kidney disease (CKD) stage 3–5. Eighty of 105 patients were explored in the bladder outcome analysis. Nadir creatinine was the strongest prognostic feature for renal function. Among features known shortly after diagnosis, the most important were baseline creatinine followed by renal dysplasia. Among tested ML models, random forest classification performed the strongest, both at 1 year of age (0.96 PR-AUC) and shortly after diagnosis (0.73 PR-AUC). Across all features combinations, unregularized logistic regression consistently demonstrated the lowest predictive performance. Bladder dysfunction was observed in 69% (55/80) of boys. However, using our ML framework, the available features did not allow reliable prediction of this outcome (around 0.82 PR-AUC against the 0.69 baseline).

**Conclusion:**

This study demonstrates that a recursive feature elimination ML framework can reliably identify the most important predictors and predict long-term renal function in boys with PUV. Baseline creatinine and renal dysplasia were identified as strong predictors and can inform parental guidance shortly after diagnosis. As expected, nadir creatinine was identified as the most powerful predictor. This study functions as a proof of concept demonstrating how ML methods can provide robust analysis of small datasets, an approach that may be valuable to other rare disease-research settings. Importantly, all models were developed using data from a single-center cohort and should undergo external validation to demonstrate generalizability.

## Introduction

Machine learning offers important advantages for prognostic modelling, also for small data analysis. Supervised ML methods are particularly well suited for clinical use, as their decision logic is generally easy to understand, which in turn promotes clinician trust and acceptance (1). They can also be tailored for clinical purposes e.g., by prioritising sensitivity when a missed diagnosis may have serious clinical consequences. Indeed, studies in pediatric diagnostic settings have shown that such recall-focused models can improve risk stratification (2). Taken together, ML frameworks that combine strong predictive performance with interpretability and transparency are well suited for clinical prediction tasks and can also provide robust analysis of small datasets in rare disease-research, such as in boys with PUV.

PUV is rare, but still the most common cause of lower urinary tract obstruction in children. It occurs only in boys, with a reported prevalence of 1.26 (1.20–1.32)/10 000 births (3). Approximately one third of these patients develop chronic kidney disease (CKD) and PUV is a leading cause of renal replacement therapy in childhood (4). Despite surgical intervention, a substantial number of patients progress to renal failure and bladder dysfunction with severe impact on health and quality of life (5–7). These patients require ongoing surveillance by both pediatricians and surgeons, and accurate prognostic assessment is important for follow-up and parental guidance.

Nadir creatinine, defined as the lowest value of creatinine measured within the first year of life or the first year after surgery, has consistently been identified as the dominant predictor of renal function in boys with PUV (8). A correlation between nadir creatinine and bladder dysfunction has also been reported (8, 9). However, reliance on nadir creatinine delays risk stratification, and the role of additional predictors remain uncertain. Prior studies have explored ML-based prediction models of urodynamic examinations (10), voiding cystourethrogram (VCUG) (11), US imaging (12) and a variety of clinical features (13), but few have focused on early predictors available shortly after diagnosis. One exception is Kim et al., who developed a logistic regression model based on clinical features available at initial presentation (14). Identifying reliable early prognostic predictors for future renal and bladder function would enable earlier detection of high-risk patients, avoid needless parental concern, and reduce unnecessary follow-up of low-risk patients.

Historically, clinical prediction models have relied on conventional statistical methods, such as unregularized logistic regression combined with expert-driven variable selection. In this study we sought to address these limitations by developing and evaluating a contemporary ML framework specifically designed for prediction in small datasets, integrating regularization, data-driven feature selection, class-imbalance handling and nested cross-validation.

Although the transformative potential of machine learning is often discussed in the context of “big data” (15, 16), far less attention has been paid to its application in “small data” settings characteristic of rare disease research (17). These datasets are typically feature-rich and imbalanced, making meaningful predictor identification particularly challenging. We therefore investigated whether a recursive feature elimination ML framework could identify the strongest early predictors of long-term renal and bladder function in boys with PUV. Importantly, the objective was not merely to compare algorithms, but to evaluate whether the ML framework presented here could improve conventional clinical prediction (18–20).

### Patients and clinical data

All boys born between 2004 and 2022 who underwent surgical treatment for PUV at our hospital were eligible for inclusion. Written parental consent was required. Patients born 2015 and later were enrolled prospectively, while data from earlier cases were obtained retrospectively from medical records. Recorded clinical data include findings at prenatal ultrasonography (US), laboratory results, VCUG, renal US, age at surgery, growth parameters, bladder function tests and history of urinary tract infections (UTI). A complete list of features included in the ML-analyses is provided in Supplementary Table S1.

## Methods

### Software and ML method specifications

All analyses were conducted in Python, using custom modules for modelling, cross validation, and data visualization. The workflow was implemented in Jupyter Notebooks, which also generated the figures.

### ML framework

**Figure 1:**
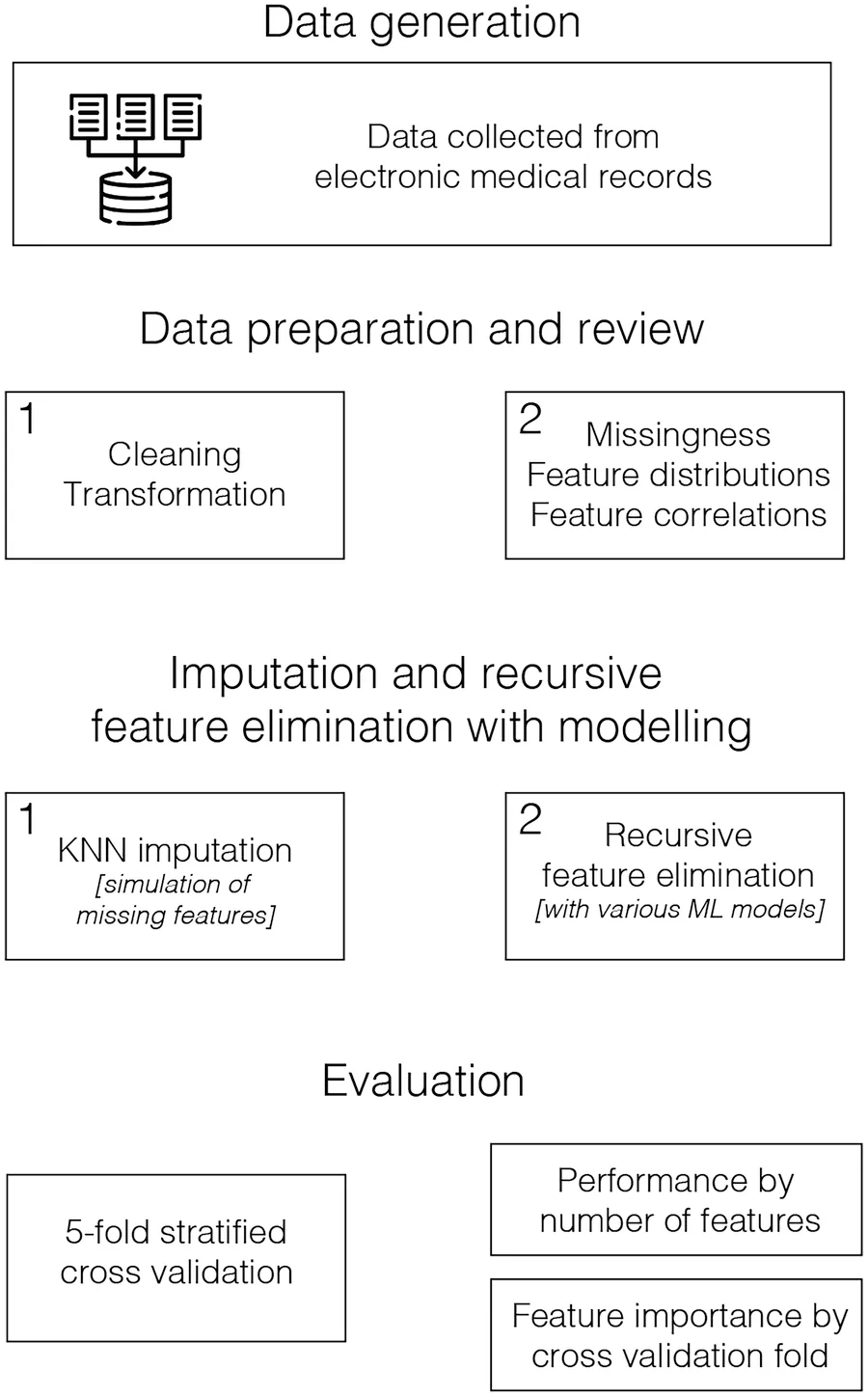
Framework of recursive feature elimination and machine learning classification. Schematic overview of the analytic flow. The process is arranged in four phases 1) data generation, 2) data preparation and review 3) imputation and recursive feature elimination with modelling and 4) evaluation.

The ML framework comprised three stages: (1) data preparation and review, (2) recursive feature elimination and modelling, and (3) evaluation (Figure 1). The framework employed a stratified cross-validation structure. Within each outer training fold, missing-value imputation (Gower-KNN), recursive feature elimination, and hyperparameter tuning were fit exclusively on the training data, while the held-out outer fold was used only for performance evaluation. Imputation was performed within the cross-validation loop rather than on the full dataset, such that the imputer was fit on each training fold only and used the training patients as neighbours to fill missing values in both the training and the held-out fold. No information from the held-out data influenced imputation. This nested design gives a more rigorous, leakage-free assessment than a single 80–20 train–test split, which in a small cohort produces unstable results that vary with the specific partition. The framework was designed both to predict renal and bladder function prognosis of boys with PUV and to determine the importance of clinical features.

### Data preparation and review

Manually curated data were cleaned, and different data types were transformed into standardized formats. This included data type checks, one-hot encoding of categorical variables, and standardising continuous variables using the training set distribution or, where available, comparable population statistics. Correlations between features were examined with Spearman correlation to spot and avoid feature redundancy. Features with completeness <75% were excluded (Supplementary Figure S1).

### Imputation

The missing values of remaining features were imputed using K-Nearest Neighbour (KNN) imputation. For each observation with missing data, the three most similar patients were identified using Gower distance, so to accommodate mixed data types. The missing value was then estimated based on the corresponding values of these nearest neighbors.

### Modelling

Four modelling approaches were compared within the ML framework: random forest, gradient boosted trees, L2 regularized logistic regression and conventional logistic regression. Two outcomes were predicted: (1) renal failure (CKD ≥ 3) at last follow-up and (2) bladder dysfunction. Additionally, single-feature logistic regression models were constructed using the top-ranked feature identified by each model.

### Recursive feature elimination

Recursive feature elimination was used to identify the most parsimonious predictive feature set. Each model was trained and tested with stratified 5-fold cross-validation using the full set of registered features, then iteratively retrained after removing the least predictive feature at each step until only one remained. Performance was assessed based on the area under the precision–recall curve (PR-AUC) across feature subset sizes. These were next assessed using repeated-measures analysis of variance (ANOVA), with the folds treated as the within-subject factor. If significant, the subset with the highest mean PR-AUC was compared with the best-performing smaller subset using a paired Wilcoxon signed-rank test (two-sided). The set with most features were retained only if they resulted in a statistically significant improvement over the parsimonious alternative.

### Evaluation and reporting

All models were evaluated using stratified five-fold cross-validation, ensuring that each fold included similar numbers of boys with renal failure or bladder dysfunction. Performance was assessed using PR-AUC across the five folds. For renal function, the performance was compared for features available at 1 year of age and those available shortly after diagnosis. Results are presented as mean ± standard error of the mean (SEM). Clinical data are listed as median values with interquartile range (IQR). Values are rounded to two decimal places, and percentages are reported as whole numbers.

### Outcomes

Chronic Kidney Disease: Estimated glomerular filtration rate (eGFR) at last follow-up was calculated using the bedside Schwartz formula. CKD was graded as described by the National Kidney Foundation Kidney Disease Outcome Quality Initiative (21, 22). Chronic renal failure is defined as eGFR < 60 mL/min/1.73 m² equivalent to CKD grade ≥ 3.

Bladder dysfunction: Bladder function was evaluated according to recommendations of the standardization committee of the international Children's Continence Society (24, 25) including clinical history, 48 h bladder diary, uroflowmetry and ultrasonographic estimates of post-void residual urine (PVR). Patients over five years of age was classified with bladder dysfunction when one or more of the following features were present:

- Daytime incontinence defined as regular episodes of urinary leakage (wet clothes or diaper/pad at least once a month)
- PVR > 20% of bladder volume (voided volume + PVR) at repeated flowmetry examinations
- Bladder emptying by use of self-catheterization (CIC)

As urinary incontinence is not applicable and flowmetry is unreliable in children under five years of age, most of the patients included in the group evaluated for bladder function were older than five at last follow-up. However, when CIC was established earlier due to poor bladder emptying, these children were also classified as having bladder dysfunction and included in the analyses of the bladder model.

### Clinical definitions

▪ Baseline-creatinine: Highest creatinine at presentation, measured at least 72 h after birth.
▪ Nadir-creatinine: Lowest creatinine in the first year of life in the early diagnosis group, or in the first year after valve ablation in the late diagnosis group.
▪ Chronic Kidney Disease (CKD): stage 3: eGFR 30–59, stage 4: eGFR 15–29 and stage 5: eGFR < 15 mL/min/1.73 m² (22).
▪ Reference values for creatinine: Day 0–14: 27–81 μmol/L, day 15–60: 21–58 μmol/L, and 2–12 months: 14–34 μmol/L.
▪ Recurrent urinary tract infections (UTI): Two or more febrile UTIs.
▪ Vesicoureteral reflux (VUR) was evaluated by a pediatric radiologist on the first VCUG prior to surgery and categorized as no VUR, unilateral- or bilateral VUR grade 3 or higher.
▪ US examinations were performed routinely during follow-up. Included in this study are US prior to surgery and at last follow-up. Renal parenchyma was described as normal or dysplastic, and categorized as normal parenchyma, unilateral- or bilateral dysplasia. Hydronephrosis was defined as anteroposterior diameter of the renal pelvis >10 mm. The urinary bladder wall was categorized by the examining pediatric radiologist as normal or thick. The presence of urinomas or urinary ascites was also registered.
▪ Z-scores were calculated for the following features: Nadir creatinine, baseline creatinine, length and weight.
▪ Bladder dysfunction was defined as incontinence and/or PRV > 20% in boys older than five years and/or in patients using self-catheterization (clean intermittent catheterization) at any age.

## Results

### Patients

A total of 107 patients were identified. Two were excluded due to lack of consent. For renal outcome analysis, patients were divided into an early diagnosis group (*n* = 83) and a late diagnosis group (*n* = 22), diagnosed before and after one year of age. For bladder outcome analysis 80 boys were eligible. The remaining 25 boys had not reached five years of age during the study period or were lost to follow-up before this age and therefore did not undergo bladder function evaluation. All patients had surgical treatment with transurethral valve resection, also referred to as valve ablation.

### Renal function

At last follow-up, 18 boys had developed chronic renal failure, defined as CKD stage 3–5, all of whom were in the early diagnosis-group. In the late diagnosis-group none of the boys developed chronic renal failure, none had elevated nadir creatinine, and only 14% (3/22) had elevated baseline creatinine. Renal outcome analysis was therefore focused on the 83 patients diagnosed before one year of age. More detailed summary statistics regarding both groups, and specifically the 7 boys with CKD stage 5, are given in Table 1.

**Table 1:** Clinical characteristics boys with PUV (not early diagnosis group).

Feature category	Features	Early diagnosis group (*n*=83)	Patients with CKD5 (*n*=7)*	Late diagnosis group (*n*=22)
Patient characteristics	Gestational age (weeks)	38.9 (36.3–40.1)	37.2 (33.9–38.3)	40 (37.3–40.3)
	Birth weight (gram)	3,400 (2,995–3,880)	3,298 (2,260–3,880)	3,260 (2,977–3,804)
	Prenatal diagnosis	56/83 (67%)	6/7 (86%)	0
	Age at diagnosis (days in early diagnosis group, years in late diagnosis group)	4 (1–20)	0	5.08 (1.84–7.59)
	Age at last follow-up (years)	9.61 (6.13–12.38)	10.97 (9.61–11.29)	10.80 (5.22–12.51)
Creatinine levels	Baseline creatinine (µmol/L)	120 (66–263)	322 (287–368)	34 (28.5–39.5)
	Nadir creatinine (µmol/L)	22 (18–33)	158 (130–199)	28 (24–36)
Ultrasound at presentation	Unilateral dysplasia	12/83 (14%)	2/7 (29%)	2/22 (9%)
	Bilateral dysplasia	18/83 (22%)	5/7 (71%)	1/22 (5%)
VCUG at presentation	Vesicoureteral reflux grade 3–5	42/83 (51%)	6/7 (86%)	3/22 (14%)
Renal function at last follow-up	Normal	65/83 (78%)	NA	22 (100%)
	CKD grade 3	10/83 (12%)	NA	0
	CKD grade 4	1/83 (1%)	NA	0
	CKD grade 5	7/83 (8%)	NA	0
Bladder assessment (*n*=80)	Daytime incontinence	36/60 (60%)	3/7 (43%)	7/20 (35%)
	CIC	4/60 (7%)	2/7 (29%)	1/20 (5%)
	Residual urine	17/60 (28%)	5/7 (71%)	7/20 (35%)
	Bladder dysfunction	45/60 (75%)	6/7 (86%)	10/20 (50%)
Renal replacement therapy (*n*=7)	Peritoneal dialysis		2/7 (29%)	
	Age at dialysis (days)		2.5 (2–3)	
	Renal transplant (Tx)		7/7 (100%)	
	Age at Tx (years)		4.44 (1.67–6.54)	

*Patients with CKD5 are also included in the early diagnosis group.

### Predictors and model performance

**Figure 2:**
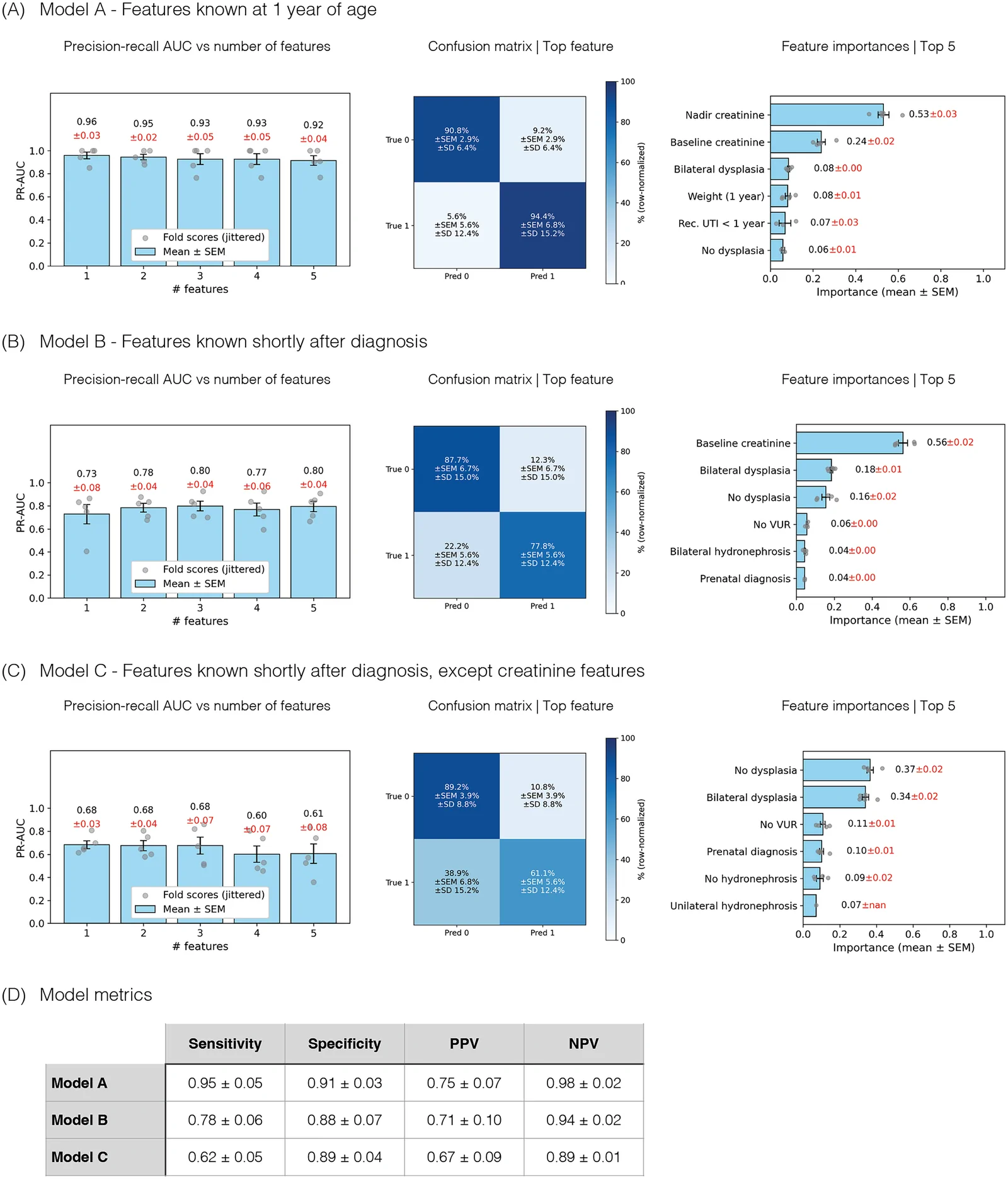
Machine learning framework for the prognostic evaluation of renal outcome in boys with PUV, using recursive feature elimination with random forest analysis (PR-AUC). Figures 2A–C: The first histogram visualizes model performance measured in mean PR-AUC (black text) with standard error (red text and error bars) across 5 cross validation folds (grey dots). In all models, the top feature alone achieved the highest PR-AUC and adding features did not improve model performance. Consequently, the final models were single feature models based on the top predictor: **A**) nadir creatinine (PR-AUC of 0.96), **B**) baseline creatinine (PR-AUC 0.73), **C**) the inverse features no dysplasia and bilateral dysplasia which showed equal importance (PR-AUC of 0.68 when either or both were included), indicating overlapping predictive value. The confusion matrix in the middle panel shows the results of the best performing models. The second histogram summarizes the five most important features. Because feature selection is performed separately in each cross-validation fold, the combined summary may include more than five features (e.g., six). Features importances sum to 100% within each fold. Error bars represent each featurés mean importance (black text) with standard error (red text) across 5 cross validation folds. Figure 2D: Overview of model performances.

**Figure 3:**
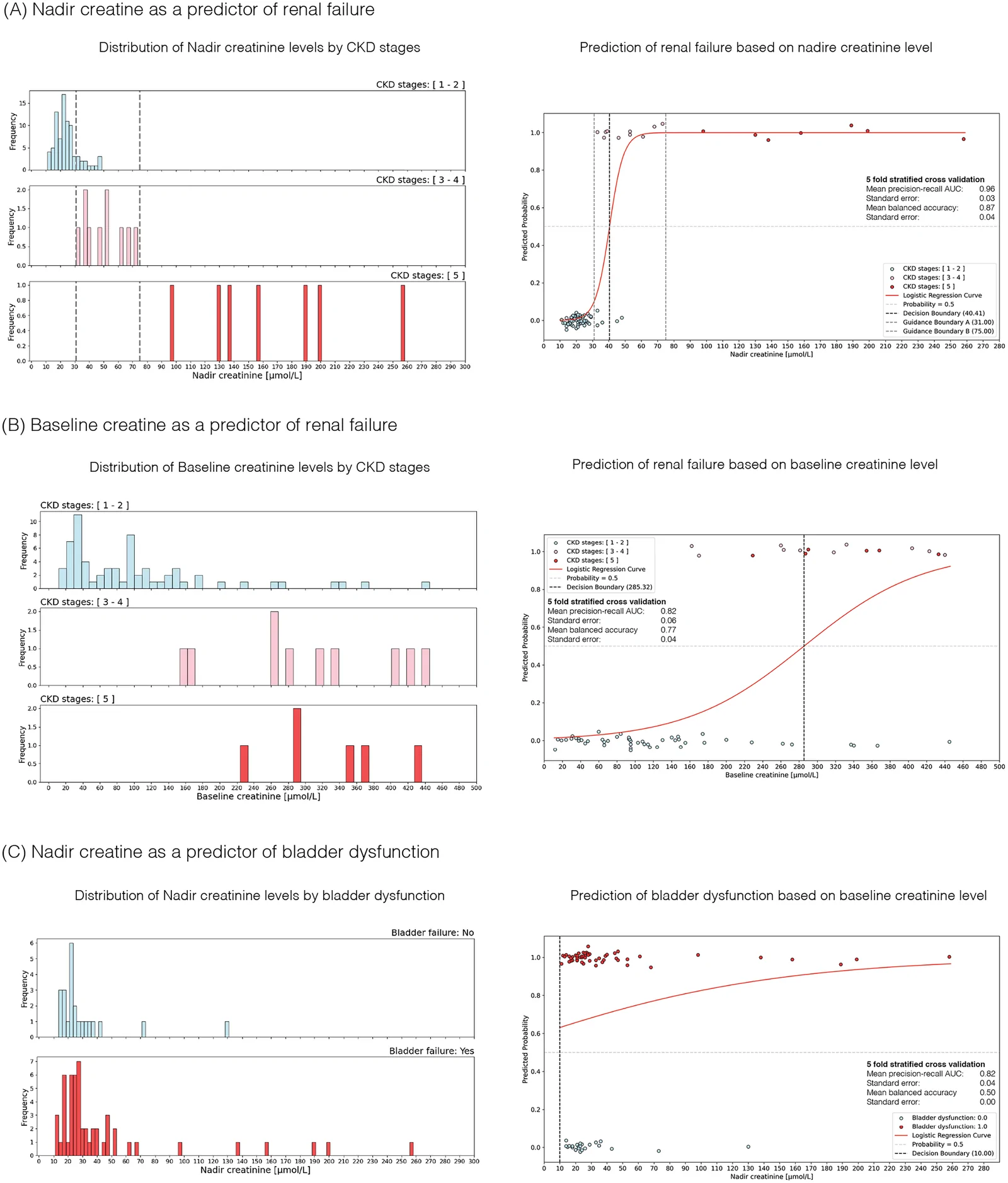
Creatinine features as sole predictors for renal and bladder outcomes in boys with PUV. The first figure shows the distribution of **A**) nadir creatinine by CKD stage, **B**) baseline creatinine by CKD stage, and **C**) nadir creatinine by bladder dysfunction. When evaluating potential cut-off values for nadir creatinine in the 105 boys with PUV, we found that among the 87 boys with normal renal function, 86 had nadir creatinine <35 µmol/L, which is within the normal range, and the last patient had nadir creatinine 45 µmol/L. Ten of eleven boys with CKD stage 3-4 had nadir creatinine of 35-75 µmol/L, while one had nadir creatinine 33 µmol/L. All 7 boys with CKD stage 5 had nadir creatinine >75 µmol/L. In Figure 3A the two grey dotted lines show previously published nadir creatinine cut-off values by Coleman et al. (36), while black dotted lines represent the models decision boundaries. For the bladder outcome analysis (Model C) 80 boys were included. The second figure presents the single-feature logistic regression models using **A**) nadir creatinine and **B**) baseline creatinine to predict future renal function, and **C**) nadir creatinine to predict future bladder dysfunction. These models achieved a mean balanced accuracy of A) 0.87, B) 0.82 and **C**) 0.5, which means that model C does not classify patients better than random guessing. For future renal failure prediction, the classification threshold was set at 50%, meaning that patients with a predicted probability of ≥ 50% of developing CKD≥3 were classified as positive. With this threshold, model A classified 3/83 boys as false positives and 4/83 boys as false negatives. Lowering the decision boundary to 30 mmol/L nadir creatinine would result in no false negatives and 13/83 false positives. Model B classified 6/83 boys as false negatives 4/83 boys as false positives, lowering the decision boundary to 160 mmol/L baseline creatinine, the model would result in no false negatives and 10/83 false positives.

Nadir creatinine was identified, among 16 features (Supplementary Figure S1), as the strongest predictor for renal function by our recursive feature elimination ML framework (Figure 2A). Shortly after diagnosis, before nadir is known, baseline creatinine was identified as the strongest (Figure 2B) among 13 features (Supplementary Figure S1). The distribution of patients by CKD stage across features is provided in Supplementary Figure S2. The random forest approach incorporating features known at 1 year of age achieved an excellent performance (mean PR-AUC of 0.96) but adding predictors beyond the dominant nadir creatinine offered no significant improvements in model performance (Figure 2A). When incorporating features available shortly after diagnosis, model performance was lower but acceptable (PR-AUC of 0.73), with additional predictors beyond baseline creatinine again providing no significant gains in model performance (Figure 2B). Excluding all creatinine values, the best early-prognosis model reached a PR-AUC of 0.68, with the inverse features no dysplasia and bilateral dysplasia emerging as the top features (Figure 2C). An overview of the distribution of CKD by both nadir and baseline creatinine is provided in Figure 3.

### Comparison of prediction models

When comparing the four approaches within the ML framework, random forest was chosen for illustration purposes, though the gradient boosting classifier and L2 regularized regression performed equally well with mean PR-AUC 0.93 and 0.96 in the model incorporating features known at 1 year of age, and PR-AUC 0.71 and 0.79 shortly after diagnosis. Unregularized conventional regression, however, achieved PR-AUCs of 0.69 at 1 year and 0.60 shortly after diagnosis (Supplementary Figure S3). An extended overview of the winning model performances is provided in Figure 2D.

### Bladder dysfunction

Fifty-five of eighty patients (69%) had bladder dysfunction. In the early diagnosis-group 45 of 60 (75%) eligible patients had bladder dysfunction and in the late diagnosis-group 10 of 20 (50%) had bladder dysfunction. Bladder dysfunction was found in 10/11 (91%) patients with CKD 3–4 and in 6/7 (86%) patients with CKD stage 5. All four modelling approaches within the ML framework performed poorly in predicting bladder dysfunction, with PR-AUCs around 0.82 against the 0.69 baseline. (Supplementary Figure S4). A single feature logistic regression model based on nadir creatinine alone illustrates that a high proportion of patients with normal nadir creatinine also develop bladder dysfunction (Figure 3C).

## Discussion

In the provided ML framework, random forest, gradient boosted trees and L2 regularized logistic regression analysis achieved high PR-AUCs in predicting long-term renal function and identifying the most parsimonious feature set needed. Conventional unregularized logistic regression analysis performed the poorest. Our ML framework identified nadir creatinine as the strongest prognostic feature and adding other features did not improve model performance, most likely due to their correlation with nadir creatinine and its superior predictive power. Shortly after diagnosis, before nadir creatinine was available, baseline creatinine was the best predictor, followed by renal dysplasia. Predicting bladder function based on the available features was not successful, with low PR-AUC for all four approaches against the baseline and a balanced accuracy of close to 0.5.

The current study demonstrates that an ML framework using recursive feature elimination can identify the most relevant predictors of renal function prognosis in boys with PUV. Consistent with previous reports, our ML framework successfully identified nadir creatinine as the most important feature among the 16 clinical features in the dataset. The ML model based solely on nadir creatinine achieved an excellent PR-AUC of 0.96. However, this renal analysis included only 18 outcome events, so a degree of overfitting cannot be excluded despite nested cross-validation. Reassuringly, performance was stable across folds and the selected predictor was consistent, but these findings require confirmation in an external cohort. The ML model based on features available shortly after diagnosis also performed well, with an PR-AUC of 0.73 including and 0.68 excluding creatinine features. These results are in line with other proposed prediction models, such as the posterior urethral valve risk of chronic kidney disease (PURK) score proposed by Kim et al., a risk stratification tool based on multivariate logistic regression analysis, which achieved a ROC-AUC (Area under the receiver operating characteristic curve) of 0.90 including features known at one year of age (14). Notably, because chronic renal failure was defined by eGFR, which derives from serum creatinine, the outcome and creatinine-based predictors share a common basis. The 1-year model's PR-AUC of 0.96 therefore confirms nadir creatinine as an established dominant predictor rather than demonstrating novel independent discrimination. Interestingly, the creatinine-excluded early model (Model C, PR-AUC 0.68), identifies bilateral renal dysplasia as the top predictor, which is independent of the outcome's creatinine basis and available shortly after diagnosis.

When comparing approaches, random forest, gradient boosted tree and L2 regularized logistic regression analysis performed equally well. The conventional unregularized logistic regression analysis performed less well (Figure 3). This is likely due to the imbalanced nature of our dataset, highlighting the role of regularization, which reduces overfitting and improves generalization by penalizing large predictor weights during training. However, the advance does not lie in the choice of algorithm, but in the framework itself, which integrates regularization, nested and stratified cross validation, recursive feature elimination, imputation, class balancing, threshold and hyperparameter optimization, and rigorous model selection. Together, these methodological components provide a robust framework for clinical prediction, illustrating how contemporary machine learning strategies can improve upon conventional approaches to clinical decision support.

ML algorithms have previously been used to develop predictive models for PUV outcomes. Kwong et al. presented an ML-model to predict deterioration of renal function based on clinical data from regular follow-up examinations (10). Their ML-model achieved a concordance-index of 0.76 for CKD progression-free survival and 0.95 for kidney replacement therapy-free survival. In their first model, renal dysplasia and baseline eGFR were most important, while nadir creatinine was the single most important feature in their second model. Kwong et al. focused on changes in renal function during follow-up, whereas our study assessed CKD as a final outcome. Both models predicted renal outcome with high accuracy, and the most important features correspond. However, where Kwong's models rely on features available from one year of age, our study adds a prospect of earlier prognostic evaluation.

Several features have been suggested as predictors of future renal function, including ultrasonographic parameters, urinary and biochemical markers and proteomic data (26, 27). However, in a review paper from 2023 by Meneghesso et al., bladder dysfunction and baseline creatinine were described as the two second most frequently reported prognostic factors after nadir creatinine (28). Since nadir creatinine is not available for several months after valve ablation, focus has been on identifying features that are available earlier. A reliable prognosis based on such early predictors would improve parental counselling, reduce emotional strain and support more personalized treatment and follow-up plans. The PURK score suggested by Kim et al. identified baseline creatinine >150 μmol/L, high grade VUR, failure to thrive and kidney dysplasia as important predictors (14). Weaver et al. used ML to compare a prognostic tool based on anatomic features from postnatal US to models based on clinical features (29). Their clinical model at one year of age (including nadir creatinine) outperformed the imaging model, while their clinical model at six months of age (excluding nadir creatinine) performed better in combination with the imaging model than either model alone. In our study, baseline creatinine and the presence or absence of bilateral renal dysplasia emerged as the strongest early predictors. Some features were less important than expected, and although previous findings suggested that the presence of urinary ascites or urinomas at prenatal US can prevent renal failure (30), these features did not seem to influence our ML model.

Previous studies report chronic renal failure in 8%–33% of boys with PUV (9, 31). While most patients who progress to end stage renal disease show signs of severe renal injury at diagnosis, chronic kidney disease may also present in later childhood or adolescence (18). In our cohort, 17% had developed CKD stage 3–5 at last follow-up, of which none were diagnosed after one year of age. Notably, this proportion is likely to increase with longer observation. Comparative studies of early versus late diagnosis report both higher, equal or lower incidence of renal failure in late diagnosed patients. These discrepancies likely reflect the broad spectrum of PUV, the complexity of the disease and differences in health-care resources. Sakin et al. found no difference between early and late diagnosed patients (32), while Hennus et al. argue that patients diagnosed later have better outcomes due to milder obstruction (9). This aligns with our finding that all boys in the late diagnosis group maintained normal renal function. In our clinical setting more severe cases tend to be detected early, while the less severe cases present later. In contrast, in low-resource settings where access to healthcare is less available, patients may be diagnosed later and more often have severe CKD at presentation (33–35).

Several attempts have been made to determine cut off-values for nadir creatinine in predicting risk of renal deterioration. A threshold of 1 mg/dL (88.4 µmol/L) is commonly used (28). Coleman et al. found in a single institution study of boys with PUV that all patients with nadir creatinine >75 µmol/L developed renal failure, while 94% of patients with nadir creatinine <35 µmol/L had normal renal function (24). Based on these findings, they proposed risk stratification into three groups: high risk (nadir creatinine >75 µmol/L), intermediate risk (nadir creatinine 35–75 µmol/L) and low risk (nadir creatinine <35 µmol/L). Our data align closely with this classification as shown in Figure 3.

Bladder dysfunction is common in boys with PUV (9), and several researchers have suggested poor functioning kidneys and bladder to be associated (36, 37). Delefortrie et al. (2022) found a significant correlation between nadir creatinine and bladder function at five years of age. In their multicenter study of 73 boys with PUV, 83% of boys with nadir creatinine >75 µmol/L developed bladder dysfunction. In boys with normal (< 35 µmol/L) or moderately increased (35–75 µmol/L) nadir creatinine, only 41% and 46% had bladder dysfunction (8). Our data supports a high prevalence of bladder dysfunction as 55 of 80 (69%) eligible boys met the criteria, and 16 of 18 (89%) boys with CKD had poor functioning bladders. However, a high proportion of patients with normal nadir creatinine also had bladder dysfunction (Figure 3C). The logistic regression model here reached a PR-AUC of 0.82 but a balanced accuracy of only 0.50. These numbers are consistent because bladder dysfunction is the majority outcome (prevalence 0.69). Hence, the no-skill PR-AUC baseline is 0.69 and 0.82 reflects the high base rate rather than genuine discrimination. The balanced accuracy of 0.50 confirms the model classifies no better than chance. The natural history of the “valve bladder syndrome” has been described as a thick-walled, overactive and noncompliant bladder in early childhood developing into chronic overdistension and poor bladder emptying in adolescence/adult life. Both stages carry risk of high bladder pressure which may cause dilatation of the upper urinary tract and influence renal function. Therefore, although the combined definition of bladder dysfunction (incontinence and/or PVR and/or CIC) may contribute to a weaker predictive performance of the ML-model compared to focusing on a single outcome, such as incontinence alone, both storage and emptying aspects of the bladder are clinically important.

Nadir creatinine is already well established as a strong predictor of renal function, but our ML models B and C identified baseline creatinine and bilateral dysplasia as well performing predictors available shortly after diagnosis. Based on the renal analysis, a risk adapted follow-up strategy could be considered, with increased follow-up of boys with high baseline creatinine and bilateral renal dysplasia and less intensive follow up of those with low baseline creatinine and no evidence of renal dysplasia. However, as this study also demonstrate limited predictability of bladder dysfunction, long-term follow up of all children who have undergone surgery for PUV is warranted.

This study demonstrates that predicative “small data” analysis is feasible, and its findings adhere to clinical experience and previous research. To make full use of a small cohort, missing values were imputed rather than excluding cases by listwise deletion. This preserves sample size and avoids discarding partially recorded patients. Given the scarcity of health data in rare pediatric diseases, synthetic data generation may represent the fastest and most practical way of obtaining adequate data for ML model development in these populations (39). Another key advantage of the recursive feature elimination ML framework is its ability to identify and rank the most important and parsimonious feature set. In this study, nadir creatinine emerged as the strongest predictor, an already well-established finding, and thus does not represent a novel discovery. However, the analysis clearly demonstrates its relative importance compared with other variables and also provides early predictors that could be useful for parental guidance. More broadly, the methods applied in this paper serves as a proof of concept, and similar methods could be valuable for clinical evaluation in other rare pediatric conditions in the future.

This single-institution study is limited by its relatively small sample size and CKD stages 3–5 were analysed as a single outcome due to the limited number of cases. Consequently, the model could not distinguish between different severities of advanced kidney disease despite the substantial variation in clinical consequences across these stages. However, as both complications of kidney disease and the risk of progression to end-stage renal disease increase significantly when GFR falls below 60 mL/min/m^2^, this outcome is important when assessing future health problems in boys with PUV. CKD ≥ 3 is also used as a primary outcome in other comparable studies of prognostic tools, including the PURK score.

Another limitation is the lack of external validation, and greater reliability would be achieved if other institutions applied the same ML framework and pooled their results. Importantly, the suggested framework is a dynamic model, and can be updated as new data becomes available, increasing its potential utility over time. To facilitate this, we have made our codebase freely available for interested clinicians or researchers who wish to update the framework with their own PUV cohort or apply it to entirely different populations, see https://github.com/g-tarrason-risa/puv-rfe-framework. Another potential direction for future research is the integration of novel biomarkers derived from blood or urine samples into the ML pipeline. This could involve evaluating the prognostic value of candidate biomarkers identified through metabolomic or genomic profiling of patients with PUV. To date, most ML models, including ours, have relied on traditional clinical features. However, the incorporation of emerging molecular biomarkers may enhance predictive performance and facilitate earlier diagnostic and prognostic assessment, particularly in the neonatal or even prenatal setting.

## Data Availability

The raw data supporting the conclusions of this article will be made available by the authors upon request. Any requests will be forwarded to the Data Protection Officer and the Ethical Committee for legal- and ethical evaluation. The data will be available for 10 years after publication and if the requesting institution has implemented the European GDPR or is able to sign the Standard Contractual Clauses for international transfers, the process will take < 6 weeks. Otherwise, inter-institutional negotiation is necessary which may prolong the wait time.

## Supplementary material

SUPPLEMENTARY FIGURE S1 Prognostic analysis of boys with PUV, included features and completeness overview in the renal sets and bladder set. A) This figure presents which features were included in the renal and bladder outcome analysis. B) This figure shows the completeness of the included features. Features with a completeness of <75% were excluded. The missing values of remaining features were imputed using K-Nearest Neighbour (KNN) imputation.

Supplementary Figure S2 Prognostic analysis of boys with PUV, complete distribution of all included features at 1 year of age by CKD stage. Overview of A) continuous and B) categorical features.

SUPPLEMENTARY FIGURE S3 Machine learning framework for the prognostic evaluation of renal outcome in boys with PUV, using three other ML models (PR-AUC). This figure shows the performance of the remaining three ML methods: gradient boosted classification, L2 regularised logistic regression, and conventional unregularized logistic regression.

SUPPLEMENTARY FIGURE S4 Machine learning framework for the prognostic evaluation of bladder outcome in boys with PUV, model performances (PR-AUC). Overview of PR-AUC and ROC-AUC model performances by feature count, using (A) a random forest classifier, (B) a gradient boosted classifier, (C) L2 regularised logistic regression and (D)conventional logistic regression. The no-skill PR-AUC baseline is 0.69 (positive class prevalence).

SUPPLEMENTARY FIGURE S5 Machine learning framework for the prognostic evaluation of renal outcome in boys with PUV, using recursive feature elimination with random forest (ROC-AUC). The histograms in A-C mirror those in Figure 2. However, instead of PR-AUC, here the performance of the random forest model is measured in mean ROC-AUC (black text) with standard error (red text and error bars) across 5 cross validation folds (grey dots), across 1 to 5 features.
